# Relationship between Respiratory Tract Complaints, Functional Status, and Smoking in Hairdressers, Auto Painters, and Carpenters

**DOI:** 10.1155/2014/802705

**Published:** 2014-07-03

**Authors:** Ümran Toru, Peri Meram Arbak, Kezban Özmen Süner, Özlem Yavuz, Naciye Karataş

**Affiliations:** ^1^Department of Chest Diseases, Dumlupınar University School of Medicine, 43100 Kütahya, Turkey; ^2^Department of Chest Diseases, Duzce University School of Medicine, 81000 Düzce, Turkey; ^3^Department of Critical Care, Hacettepe University School of Medicine, 06100 Ankara, Turkey; ^4^Department of Biochemistry, Balıkesir University School of Medicine, 10145 Balıkesir, Turkey; ^5^Department of Chest Diseases, Antakya State Hospital, 3100 Antakya, Turkey

## Abstract

*Background and Aim*. It was observed that occupation and smoking increased each other's effects on the development of airway diseases. We aimed to search the relationship between respiratory symptoms, smoking, and occupation. *Materials and Methods*. 225 employees in Düzce, Turkey, were applied a survey questioning respiratory complaints, pulmonary function tests (PFTs) and cotinine measurements in urine. *Results*. Cough (26.7%), phlegm (30.7%), and chest tightness (21.3%) were encountered more in carpenters compared to other groups and phlegm was statistically higher at significant level compared to other groups. The complaints of cough (30.4%), phlegm (27.4%), and chest tightness (21.5%) were significantly higher in individuals whose cotinine level was above 500 ng/mL and forced expiratory volume in one second (FEV1)/forced vital capacity (FVC) ratio, maximum midexpiratory flow rate (MMFR) values were significantly lower. Dyspnea complaint of auto painters whose cotinine level was below 500 ng/mL was significantly higher and also expected MMFR% value of this group was significantly lower compared to other groups. While age had independent effect on respiratory function tests, type of the job was found to be independently effective on MMFR. *Conclusion*. Smoking increases respiratory complaints of employees. In auto painters, the occupation causes airway disease regardless of smoking.

## 1. Introduction

It is a subject of various researches whether smoking directly causes occupational lung diseases or has indirect consequences. While smoking causes sensitivity to certain factors, it sometimes increases the risk of occupational asthma (OA) development by means of certain sensitizer agents [1–8]. It has been demonstrated that there is low serum IgG and high IgE in smokers [9]. In a study by McSharry et al., it was shown that the main factor which seemed to determine the antibody isotype response was cigarette smoking. IgE antibody was produced mainly by smokers, whereas IgG antibody was the predominant isotype produced by nonsmokers [10]. The interaction with inhaled allergen in nonsmokers causes IgG antibody development and extrinsic allergic alveolitis and this is the evidence that smoking changes immunological response [11].

In certain studies, it was reported that smoking increases sensitivity risk with platinum salts, green coffee, Indian beans, shrimps, and flour [10, 12–16]. The studies for acid anhydrite exposure found out both negative and positive correlations with specific IgE [17, 18]. A study demonstrated the increased sensitivity risk in bakers; however, smoking did not increase the asthma risk in bakers. It was shown that smoking increases the risk of sensitivity against high-molecular-weight agents causing occupational asthma by means of IgE mediated mechanism [13, 19–21].

In this study, it was aimed to study respiratory complaints and smoking frequency as well as smoking and occupational and respiratory complaint frequency in various occupational groups (those exposed to wood/tree dust, car paint, and hair dyes). Consequently, the study will reveal the frequency of respiratory complaints observed in such occupational groups and the relevance of such frequency with smoking and also with the occupation itself.

## 2. Materials and Methods

The study was conducted during the months of April, May, June, and July in 2009 in the province of Düzce (a province situated in the northwestern part of Turkey with a central population of 130.000 whose economy is based on agriculture and small-scale industry).

The study group consisted of two hundred twenty-five employees working in different branches of small and medium enterprises (SMEs) of the province Düzce. The records of enterprises that were to comprise the study group were obtained from the Chamber of Artisans and Craftsmen of the province. The hairdressers, carpenters, and car painting workshops to be included in the study were selected randomly. Respiratory survey, physical examination, and pulmonary function tests were performed within working hours in the business places. The urine samples that were obtained within working hours for urine cotinine levels were measured by studying with cotinine kits in laboratory environment. The study was arranged as cross-sectional.

The individuals with a history of upper respiratory tract infection within the last one month or with a disease diagnosis that was known based on medical history and that might cause dyspnea (heart failure, bronchiectasis, COPD, etc.) were not included in the study. All of the individuals that took part in the study were informed about the purpose of the study and each participant was asked to give his/her written consent about taking part in the study voluntarily. The study was approved by the Ethic Board of Medicine Faculty of Düzce University.

### 2.1. Survey

The individuals were applied face-to-face survey within working hours by the same doctor. The survey was a modified form of American Thoracic Society (ATS) respiratory disease survey. Survey questions included age, gender, place of survey, date, occupational history, exposure to chemical substances, curriculum vitae (other respiratory disease history), presence of ventilation at work site, use of mask at the work site, presence of respiratory symptoms (cough, phlegm, wheezing, chest tightness, dyspnea, exertional dyspnea, and hemoptysis), occupation relationship, smoking status, farming and animal feeding history, asthma-allergy history before the job, allergic indications (conjunctivitis, rhinitis, etc.), and occupation relationship.

### 2.2. Physical Examination

Physical examination was conducted by the same doctor in business place. During the examination of chest, chest deformity, back and front diameter of chest, vibration thoracic, expansion, sonority, rale, rhonchus, and wheezy respiration were examined.

### 2.3. Spirometric Measurements

In spirometric measurements, dry portable spirometer (Vitalograph ALPHA) was used. These measurements were performed as per the American Thoracic Society (ATS) standards. The participants were asked to do respiratory maneuvers closing their noses by themselves in seated position in the working place. Deep expiratory application after deep inspirium was repeated at least three times and the best measurement was recorded. Pulmonary function test (PFT) was applied by the same doctor. Forced expiratory flow-volume parameters were studied. Forced vital capacity (FVC), forced expiratory volume in one second (FEV1), Tiffeneau index (FEV1/FVC), maximum midexpiratory flow rate (MMFR), or forced expiratory flow between 25 percent and 75 percent of vital capacity (FEF (25–75)) values were used in the tables. PFTs were performed once during the working. Measurements were performed in the months of April, May, June, and July in 2009.

### 2.4. Urine Cotinine Measurement

Urine samples were received during working hours for urine cotinine measurement. These samples were examined with cotinine kits called “Immulite 1000 Nicotine Metabolite” of Siemens in laboratory environment and urine cotinine levels were determined. Cotinine level cutoff value was 500 ng/mL. The individuals whose cotinine level was below 500 ng/mL were not active smokers.

### 2.5. Statistics

The features of the study group were decided using Chi-square test and Student's *t*-test on SPSS 13-0 software. The parametrical values of three groups were evaluated using one-way ANOVA while subgroup comparisons were performed with Bonferroni test. In order to determine the factors with independent effects on respiratory complaints and rhinitis, univariant and multivariant analyses were implemented. In order to determine the factors that had independent effects on respiratory function parameters, linear regression analysis was applied. *P* value was taken as <0.05 for statistical significance.

## 3. Results

There was significant difference between female/male distribution in three groups. While most of the hairdressers were female (68/7), the majority of auto painters (−/75) and carpenters (4/71) were male. Average age values were 27.2 ± 9.2 years (min 15–max 52) in hairdressers, 36.9 ± 10.3 years (min 14–max 73) in auto painters, and 32.4 ± 9.7 years (min 16–max 56) in carpenters (*P* = 0.0003). The results of respiratory function tests and frequency of respiratory symptoms in cases are shown in Table 1.

Cough, phlegm, and chest tightness were higher in carpenters compared to other groups and phlegm was statistically and significantly higher (*P* = 0.017). Dyspnea was higher in auto painters compared to other groups (*P* = 0.029). FEV1 and MMFR were found to be significantly lower in hairdressers compared to other groups (*P* = 0.005, *P* = 0.001). The working period of auto painters was significantly longer compared to other groups (*P* = 0.0001).

While smoking rate was the highest among auto painters (56/75), hairdressers (42/75) and carpenters (43/75) demonstrated a similar trend too. Nonsmoking rates were highest among hairdressers (29/75) and carpenters (24/75); the number of nonsmoking auto painters was fewer (11/74). Four hairdressers, 8 auto painters, and 8 carpenters were ex-smokers. Mean ages of subjects with high (135 subjects) and low cotinine (90 subjects) levels were 33.1 ± 9.4 and 30.9 ± 11.8, respectively (*P* = 0.155). Respiratory tract complaints and respiratory function tests according to smoking status are shown in Table 2.

Cough, phlegm, and chest tightness were significantly more in individuals whose cotinine level was above 500 ng/mL. FEV1/FVC ratio and MMFR value were significantly lower (*P* = 0.008, *P* = 0.033).

**Figure 1:**
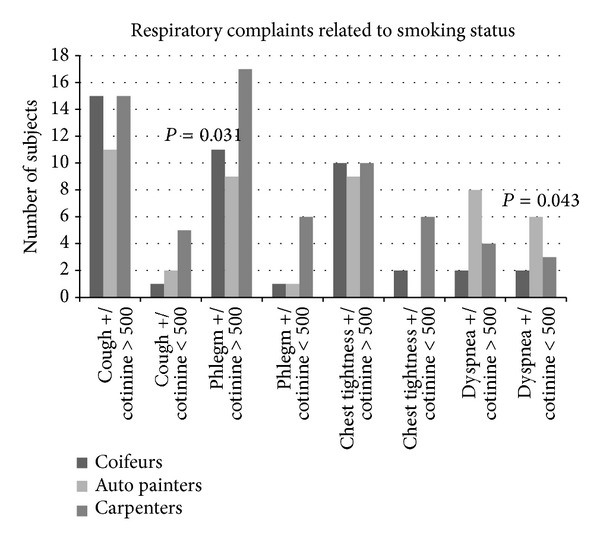
Relationship between number of workers and respiratory symptoms related to smoking (Chi-square test was used to compare the rates of complaints of groups).

The rates of cough, phlegm, chest tightness, and dyspnea in three groups whose urine cotinine level was above or below 500 ng/mL are shown in Figure 1.

There was more cough complaints in carpenters' group, whose cotinine level was below 500 ng/mL, compared to other groups; however, there was no statistically significant difference between the three groups. There was no significant difference between the three groups in respect of the presence of cough whose cotinine level was above 500 ng/mL.

There was no significant difference in respect of phlegm between the three groups whose cotinine level was below 500 ng/mL. Phlegm was significantly higher in carpenters whose cotinine level was above 500 ng/mL compared to other groups (*P* = 0.031).

Even though chest tightness was felt more in carpenters whose cotinine level was below 500 ng/mL compared to other groups, the differences were not statistically significant. There was no difference in respect of chest tightness in those groups whose cotinine level was above 500 ng/mL.

**Figure 2:**
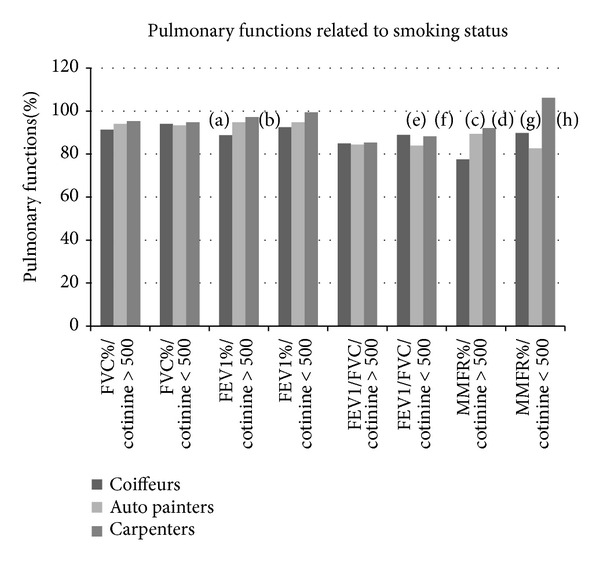
Relationship between mean predicted values and pulmonary functions related to smoking (one-way ANOVA was used to compare groups). (a) % FEV1 of coiffeurs < % FEV1 of auto painters (*P* = 0.032). (b) % FEV1 of coiffeurs < % FEV1 of carpenters (*P* = 0.004). (c) % MMFR of coiffeurs < %MMFR of auto painters (*P* = 0.018). (d) % MMFR of coiffeurs < % MMFR of carpenters (*P* = 0.006). (e) FEV1/FVC of auto painters < FEV1/FVC of coiffeurs (*P* = 0.013). (f) FEV1/FVC of auto painters < FEV1/FVC of carpenters (*P* = 0.034). (g) % MMFR of coiffeurs < % MMFR of carpenters (*P* = 0.017). (h) % MMFR of auto painters < % MMFR of carpenters (*P* = 0.003).

The rate of dyspnea in auto painters whose cotinine level was below 500 ng/mL was significantly higher compared to other groups (*P* = 0.043). Even though dyspnea was encountered more in auto painters whose cotinine level was above 500 ng/mL compared to other groups, the difference was not significant. The average expected FVC, FEV1, and MMFR values of employees whose cotinine level was below 500 ng/mL are shown in Figure 2.

There was no significant difference between average FVC values of the three occupational groups. Expected average % FEV1 value among hairdressers, in which cotinine value was above 500 ng/mL, was significantly lower than the other two groups (*P* = 0.013). Expected average % FEV1/FVC value among auto painters, in which cotinine value was below 500 ng/mL, was significantly lower than the other two groups (*P* = 0.035). Expected average % MMFR value among hairdressers, in which cotinine value was above 500 ng/mL, was significantly lower than the other two groups (*P* = 0.013). Expected average % MMFR value among auto painters, in which cotinine value was below 500 ng/mL, was significantly lower than other two groups (*P* = 0.006).

Even though rhinitis was observed more frequently in hairdressers (8/41) whose cotinine level was above 500 ng/mL compared to other groups (auto painters: 3/53, carpenters: 4/41), the difference was not significant. Rhinitis was encountered at similar frequency in the three occupational groups whose cotinine level was below 500 ng/mL (2 of 34 coiffeurs, 4 of 22 auto painters, and 3 of 34 carpenters had rhinitis).

In univariant analysis, smoking was found to be independently effective on phlegm (mean square = 0.556, *F* = 4.360, *P* = 0.016), wheezing (mean square = 0.616, *F* = 3.749, *P* = 0.027), and dyspnea (mean square = 0.737, *F* = 10.438, *P* = 0.0008) significantly. Working duration was independently effective on dyspnea (mean square = 0.123, *F* = 1.741, *P* = 0.017). Independent effects of smoking on phlegm (mean square = 0.843, *F* = 6.304, *P* = 0.002), wheezing (mean square = 0.667, *F* = 4.131, *P* = 0.018), and dyspnea (mean square = 0.728, *F* = 7.941, *P* = 0.001) were confirmed with multivariate analysis.

While age was found to be independently effective on % predicted FVC (beta = −0.337, *t* = −2.661, *P* = 0.008), FEV1 (beta = −0.367, *t* = −3.002, *P* = 0.003), FEV1/FVC (beta = −0.454, *t* = −3.802, *P* = 0.0002), and MMFR (beta = −0.372, *t* = −3.094, *P* = 0.002), job was found to be independently effective on FEV1 (beta = 0.211, *t* = 2.007, *P* = 0.046) and MMFR (beta = 0.245, *t* = 2.362, *P* = 0.019).

## 4. Discussion

Hairdressers, auto painters, and carpenters are occupational groups that are frequently found in almost all regions of Turkey and occupational airway diseases are encountered in these groups. Smoking may aggravate occupational airway disease in addition to dust, smoke, and steams exposed in working places. In our study, it was observed that smoking both increased respiratory complaints and decreased respiratory functions in all three occupational groups. However, dyspnea and rhinitis indications did not differ based on smoking status.

In hairdressers respiratory disease prevalence and occupational asthma risk increased. In certain studies, it was demonstrated that major agents that caused occupational asthma and occupational rhinitis in hairdressers were persulphate salts. Hairdressers are at risk in respect of occupational respiratory diseases; however, risk factors, causing agents, and underlining mechanisms could not be defined exactly [22–24].

In a study by Brisman et al. that was performed with the aim of evaluating the wheezing, dry cough, and nasal congestion in hairdressers, the survey on respiratory symptoms, atopy, smoking, and job history was answered by three thousand nine hundred and fifty-seven female hairdressers and four thousand nine hundred and five reference women from general population. When compared with references, the ratios of all three symptoms in hairdressers were found to be high and hairdressing was correlated with an increased incidence of respiratory symptoms. The combined effect of hairdressing and smoking was found to be lower than expected and it was proposed that smoking has negative modifying effect [25].

In a study by Slater et al., the occupational respiratory symptoms in hairdressers were examined. Employees of a hundred hairdressers and one hundred and six offices and stores were applied a survey covering their respiratory symptoms, demographic data, and smoking habits and respiratory functions were measured before each shift. Asthma symptoms, asthma diagnosis, and asthma attack prevalence of hairdressers in the previous twelve-month period were found to be high; however, it was found out that these differences significantly decreased after being corrected by age, gender, and smoking habits. High symptom prevalence in hairdressers was associated with high smoking ratios and average pulmonary function values were found to be low [26].

Cough, phlegm, and chest tightness were observed more in smokers compared to nonsmokers in accordance with the literature in our study. Rhinitis was more in smoking hairdressers. It was determined that rhinitis was encountered the most in hairdressers (13.3%) compared to auto painters (9.3%) and carpenters (9.3%). In the comparison between smokers, rhinitis was encountered the most among hairdressers. Akpinar-Elci et al. observed significant occupational asthma risk among hairdressers and reported occupational asthma prevalence as 14.6%. Furthermore, they observed increased risk for occupational asthma with allergic rhinitis and conjunctivitis [27]. MMFR average, which is an indicator of small-medium airway obstruction, was found to be higher in hairdressers compared to auto painters but lower compared to carpenters with the cotinine levels below 500 ng/mL. Chronic inhalation of hair polishers can account for the bronchial irritation and obstruction of small airways among hairdressers [28].

In a study by Parra et al., it was shown that potassium and sodium persulphate extracts of hair bleaches caused late developing asthmatic reaction [29].

Wood dust contains so many microorganisms (including fungus), toxins, and chemical substances and these can affect human health significantly. It has been reported that these agents cause irritation in oral cavity and throat, chest tightness, irritant dermatitis, urticaria, alveolitis, and deterioration in pulmonary functions and reduction in FEV1 [30–34].

In a study by Shamssain, it was observed that especially cough and nasal symptoms in employees who worked in furniture factories and exposed to wood dust increased as the number of working years increased. In the same study, it was found out that FVC decreased by 26 mL for each year [35].

In a study by Carosso et al. on wood workers and healthy control group without any exposure, it was stressed that exposure to wood dust or certain bronchoreactive substances related to wood processing could induce COPD and certain wood dust asthma cases were found to be related to suddenly developing allergic reaction [36].

In this study, chronic bronchitis symptoms such as cough (26.7%) and phlegm (30.7%) in carpenters were found to be more frequent compared to hairdressers and auto painters and increase in phlegm was statistically significant. This was in line with studies which reported that exposure to wood dust could be related to COPD. In a study by Jacobsen et al., an accelerated decrease in annual FEV1 ratio was found in female wood workers—especially in smokers—compared to reference workers [37].

In our study, phlegm was more frequently observed in carpenters whose cotinine level was above 500 ng/mL. And this made the researchers think if smoking disturbed respiratory functions in addition to occupational exposure. The expected FVC, FEV1, and MMFR values in carpenters were higher than hairdressers and auto painters and such difference was statistically significant for FEV1 and MMFR. All expiratory flow values of carpenters whose cotinine level was below 500 ng/mL were higher than hairdressers and auto painters. It was observed that respiratory functions of carpenters were protected better than the other two groups when there was no smoking.

Diisocyanates are highly reactive monomers that are the most widespread cause of occupational asthma. Exposure to these substances is quite frequent in auto painting business that is very predominant in Turkey. In a study by Cullen et al., airway symptoms in line with occupational asthma in auto painters were highly observed (19.6%). Smoking seems to be correlated with symptoms without being associated with atopic risk. It was observed that regular use of respirators that enable air exchange was associated with lower risk in workers that performed painting part time or full time [2].

In the study of Ucgun et al., 30 workers (9.6%) received OA diagnosis upon survey, typical history, PFT values, peak expiratory flow (PEF) monitoring, and nonspecific bronchial provocation tests (NSBPT). When smoking habits and atopy in workers with OA diagnosis were compared to other workers, they were found to be statistically and significantly high. It was emphasized that OA is a frequent disease in car and furniture painters and smoking habits and atopy have a significant effect on the development of OA [7].

In this study, dyspnea rate was higher in auto painters compared to hairdressers and carpenters (*P* = 0.029). Expected percentages of FVC, FEV1, FEV1/FVC ratio, and MMFR values in auto painters were lower than values of carpenters. Since both occupational groups consisted of men dominantly, it was considered that this could be due to higher rate of smoking, longer average working period, and more occupational exposure among auto painters than carpenters whose respiratory functions are affected worse. When respiratory complaints were evaluated according to cotinine level, all complaints (cough, phlegm, and chest tightness) apart from dyspnea in auto painters were higher in those with cotinine level above 500 ng/mL. Dyspnea was observed at similar levels in smoker and nonsmoker auto painters. Expected percentage of MMFR and FEV1/FVC values of nonsmoker auto painters were significantly lower compared to nonsmoker hairdressers and carpenters.

In regression analysis, job was found to be independently effective on % FEV1 and MMFR. And this made the researchers think that occupational exposure had the greatest effect on auto painters.

The limitations of our study were application of survey specific to OA, measurement of PEF rates in suitable cases, and nonpresence of skin tests and nasal throat examination.

In conclusion, hairdressers were determined as an occupational group the majority of which was formed by women, in which respiratory complaints and rhinitis history were frequently observed in the subgroup in which cotinine was above 500 ng/mL and where both occupational and smoking status had an additive effect. Carpenters demonstrated symptoms in line with chronic bronchitis and this was more prominent in carpenters whose cotinine level was above 500 ng/mL. However, carpenters attracted more attention by better protection of respiratory function tests than hairdressers and auto painters.

Rhinitis history was found to be lower in carpenters than hairdressers. This made the researchers think that COPD could be observed in long-term follow-up in carpenters and this would increase with the smoking status. Dyspnea rate was higher in auto painters compared to hairdressers and carpenters. Expected percentage of FVC, FEV1, FEV1/FVC ratio, and MMFR values of auto painters were lower than values of carpenters. Low levels of FEV1/FVC and MMFR in nonsmoking subgroup of auto painters were considered to be related to occupation.

Evaluation of individuals in three occupational groups that are widespread in our country in respect of smoking and occupational features on respiratory complaints and functions presented invaluable information in respect of protecting the respiratory health in these occupational groups.

## 5. Conclusions

Smoking increases respiratory complaints of employees. Cutting off smoking and use of respiratory protective equipment and also ensuring dust and smoke control are quite important.

## Figures and Tables

**Table 1:** Respiratory symptoms and respiratory function tests of cases.

	Hairdressers *n* = 75	Auto painters *n* = 75	Carpenters *n* = 75	*P*
Cough (*n*/%)	16 (21.3)	13 (17.3)	20 (26.7)	0.381
Phlegm (*n*/%)	12 (16.0)	10 (13.3)	23 (30.7)	0.017
Chest tightness (*n*/%)	12 (16.0)	9 (12.0)	16 (21.3)	0.302
Dyspnea (*n*/%)	4 (5.3)	14 (18.7)	7 (9.3)	0.029
Rhinitis (*n*/%)	10 (13.3)	7 (9.3)	7 (9.3)	0.657
FVC/SD (%predicted)	92.5 ± 14.5	93.9 ± 13.0	95.1 ± 12.7	0.512
FEV1/SD (%predicted)	90.5 ± 13.6	94.7 ± 14.2	98.2 ± 14.9	0.005
FEV1/FVC (%predicted)	86.8 ± 6.7	84.3 ± 8.0	86.7 ± 6.5	0.052
MMFR/SD (%predicted)	83.1 ± 22.7	87.4 ± 24.6	98.4 ± 29.3	0.001
Working duration (years)	11.3 ± 9.1	22.1 ± 9.8	11.4 ± 8.8	0.0001

**Table 2:** Respiratory tract complaints and respiratory function tests according to smoking status.

	Cotinine levels above 500 ng/mL	Cotinine levels below 500 ng/mL	*P*
Cough	41 (30.4%)	8 (8.9%)	0.000
Phlegm	37 (27.4%)	8 (8.9%)	0.003
Chest tightness	29 (21.5%)	8 (8.9%)	0.009
Dyspnea	14 (10.4%)	11 (12.2%)	0.410
Rhinitis	15 (11.1%)	9 (10.0%)	0.487
FVC	93.6 ± 12.2	94.1 ± 15.0	0.777
FEV1	93.7 ± 13.4	95.7 ± 16.0	0.317
FEV1/FVC	84.9 ± 6.7	87.4 ± 7.6	0.008
MMFR	86.6 ± 24.1	94.2 ± 29.0	0.033
